# Energy, Macronutrient Intake, and Anthropometrics of Vegetarian, Vegan, and Omnivorous Children (1–3 Years) in Germany (VeChi Diet Study)

**DOI:** 10.3390/nu11040832

**Published:** 2019-04-12

**Authors:** Stine Weder, Morwenna Hoffmann, Katja Becker, Ute Alexy, Markus Keller

**Affiliations:** 1 Fachhochschule des Mittelstands (FHM), University of Applied Sciences, 33602 Bielefeld, Germany; weder@fh-mittelstand.de (S.W.); morwenna.hoffmann@fh-mittelstand.de (M.H.); 2 Biochemistry and Molecular Biology, Interdisciplinary Research Center, Justus Liebig University Giessen, 35392 Giessen, Germany; katja.becker@uni-giessen.de; 3 IEL-Nutritional Epidemiology, DONALD Study, University of Bonn, 44225 Dortmund, Germany; alexy@uni-bonn.de

**Keywords:** vegetarian, vegan, children, energy, macronutrients, nutrient intake, body weight, body height, WHO Child Growth Standards

## Abstract

Due to the lack of current, large-scale studies examining their dietary intake and health, there are concerns about vegetarian (VG) and vegan (VN) diets in childhood. Therefore, the Vegetarian and Vegan Children Study (VeChi Diet Study) examined the energy and macronutrient intake as well as the anthropometrics of 430 VG, VN, and omnivorous (OM) children (1–3 years) in Germany. A 3-day weighed dietary record assessed dietary intake, and an online questionnaire assessed lifestyle, body weight (BW), and height. Average dietary intakes and anthropometrics were compared between groups using ANCOVA. There were no significant differences in energy intake or density and anthropometrics between the study groups. OM children had the highest adjusted median intakes of protein (OM: 2.7, VG: 2.3, VN: 2.4 g/kg BW, *p* < 0.0001), fat (OM: 36.0, VG: 33.5, VN: 31.2%E, *p* < 0.0001), and added sugars (OM: 5.3, VG: 4.5, VN: 3.8%E, *p* = 0.002), whereas VN children had the highest adjusted intakes of carbohydrates (OM: 50.1, VG: 54.1, VN: 56.2%E, *p* < 0.0001) and fiber (OM: 12.2, VG: 16.5, VN: 21.8 g/1,000 kcal, *p* < 0.0001). Therefore, a VG and VN diet in early childhood can provide the same amount of energy and macronutrients, leading to a normal growth in comparison to OM children.

## 1. Introduction

A vegetarian (VG) diet excludes meat and fish, while a vegan (VN) diet is defined by the total absence of animal foods, including dairy products and eggs. In Germany, VG and VN diets have become increasingly popular in the last several decades. The most recent estimations assume that about 2.5–10% of German adults are VGs and 0.3–1.6% are VNs [1,2,3,4,5]. Although the number of children on such plant-based diets in Germany is unknown, VG and VN parents probably also raise their children without meat or any foods of animal origin.

There is a scientific debate on whether or not VG and in particular VN diets are appropriate for children since, during growth, energy and nutrient requirements are higher than for adults relative to their body weight (BW) [6,7,8]. While the German Nutrition Society (Deutsche Gesellschaft für Ernährung, DGE) does not recommend a VN diet for infants, children, or adolescents (as well as for pregnant and lactating women) [7], the Academy of Nutrition and Dietetics (AND) from the USA stated that “well-planned VN, lacto-VG, and lacto-ovo-VG diets are appropriate for all stages of the life cycle, including pregnancy and lactation” [9,10,11]. This discrepancy is presumably caused by the lack of studies on VG and VN diets and health during childhood.

In the majority of available studies, VG and VN children showed normal growth and development, but in some studies, VG and VN children tended to be thinner and (in particular in samples <5 years of age) smaller than the reference populations [12,13]. Reference populations in those past investigations were often formula-fed infants (e.g., [14]) that tend to gain more BW and grow differently than breastfed infants [15]. The lower BW and body height (BH) of some VG and VN children therefore could at least partially be explained by the fact that VG and VN children are more likely being breastfed than non-vegetarian children [14,16,17]. However, growth retardation on VN or VG diets might be an issue of concern due to lower energy intake, protein intake, and the quality of vegetable foods. On the other hand, VG and VN adults have a lower risk of overweight and obesity [18,19]; therefore, an early VG or VN diet is discussed as suitable to prevent pediatric obesity [18,20].

There are some nutrients regarded to be critical in VG (iron, zinc, iodine, selenium, long chain *n*-3 fatty acids (eicosapentaenoic acid, docosahexaenoic acid), and vitamin D) and VN diets (additionally vitamin B_12_, calcium, vitamin B_2_, and protein) [7,9,21]. Nevertheless, in the majority of available studies, VG and VN adults meet the dietary reference intakes of macronutrients (protein, fat, and carbohydrates), many micronutrients (e.g., magnesium, folate, vitamin B_1_, biotin, pantothenic acid, vitamin C, and β-carotene), and fiber more often than OM control groups [9,16,22,23,24,25,26].

The few published studies with young VG and VN children (age 1–3 years) showed comparable results. Their nutrient intake was broadly in line with the reference values and the intake/status of micronutrients (folate, vitamin A, and vitamin C); dietary fiber was in the recommended range or even higher than the control groups and/or reference values, whereas energy, vitamin B_2_, vitamin B_12_, vitamin D, iron, and calcium were more often below the reference values and/or lower than in the OM control groups [27,28,29,30,31,32,33,34]. In some studies, there were no differences in macronutrient intake between young VG and OM children [28,31,33,35]. In other studies, young VG children had higher intakes of carbohydrates but lower intakes of fat [29,36].

However, studies on VG or VN diets during childhood are highly heterogeneous, mostly cross-sectional, of small sample sizes, and outdated (mainly from the 1970–1990s) [12,13]. Since then, due to the trend toward plant-based diets, the food market has changed and now offers an increasing number of VN or VG meat or sausage substitutes, plant-based milk alternatives, or special supplements for this population subgroup. Moreover, the World Wide Web provides a great deal of information on risks and benefits of plant-based diets and enables experience exchange among VN or VG families. However, there is an urgent need to investigate the current nutritional and health status of children on a modern VG or VN diet. One main objective of the Vegetarian and Vegan Children Study (VeChi Diet Study) therefore was to compare the intake of energy, macronutrients, and fiber, as well as BW and BH, of VG, VN, and OM children aged 1–3 years in Germany.

## 2. Materials and Methods

### 2.1. Study Design and Participants

The VeChi Diet Study is a cross-sectional study collecting data on diet, lifestyle, BW, and BH from VG, VN, and OM children (1–3 years). Subjects were recruited throughout Germany between August 2016 and March 2018 mainly via the study website (www.vechi-studie.de), VN/VG/child nutrition Facebook groups, a mailing list of Giessen University, magazines and journals, VN/VG websites, daycare centers, and VN/VG conventions. Furthermore, participating families were asked to recruit friends of their children. Inclusion criteria were VG, VN, or OM children (age 1–3 years) living in Germany. Exclusion criteria were (1) diagnosed diseases that could affect the studied variables (e.g., enteropathy, pancreatic diseases, and metabolic disorders such as phenylketonuria or fructose malabsorption) and (2) special diets other than vegan or vegetarian diets, e.g., predominantly (≥70%) raw food diet according to [37]. Parents participating with their children did not receive any financial incentive but were provided with the results of the dietary record. The observational and non-invasive study was conducted according to the guidelines of the Declaration of Helsinki and approved by the Ethics Committee of the University of Bonn (046/17). The study is registered at the German Clinical Trials Register (DRKS00010982). All examinations are performed with parental written consent.

Since the recruitment procedure did not reveal a sufficient number of OM participants, data from participants of the DONALD (DOrtmund Nutritional and Anthropometric Longitudinally Designed) study who met the same inclusion criteria as the VeChi Diet Study (1–3 years, healthy, living in Germany) were also included. The DONALD study is an ongoing cohort study that started in 1985 to collect information on diet, growth, development, and metabolism of healthy children and adolescents in Dortmund, Germany. Yearly examinations include 3-day weighed dietary records, anthropometric measurements, and interviews on lifestyle. The Ethics Committee of the University of Bonn approved the study, and all examinations are performed with parental and later with children’s written consent [38]. The parents of DONALD participants whose data were used in the VeChi Diet Study were additionally asked questions that are not included in the regular DONALD study protocol.

### 2.2. Sample Size Estimation

As no published data were available for power estimations, dietary intake data from the DONALD study (tertiles of meat intake, covariates age and sex) were used for sample size calculations. A partial correlation between predictor (diet category; here tertiles of meat intake) and outcome (food, energy, and nutrient intake; here: energy, protein, vitamin B_12_, and zinc intake) of 0.2 was likely. This resulted in a predicted power of 0.97 and an estimated required total sample size of 450 children. Because the correlation between zero meat intake and the outcomes had been expected to be even greater, a sample size of 430 was assumed sufficient enough to detect the expected statistic differences.

### 2.3. Data Assessment

Data were collected from October 2016 to April 2018.

#### 2.3.1. Nutrition Assessment

Dietary intake in the VeChi Diet Study was assessed using 3-day weighed dietary records in accordance with the procedure of the DONALD study [38]. The parents weighed and recorded all foods and beverages consumed by the participating children, as well as leftovers, over three consecutive days (weekdays and weekends) using electronic kitchen scales. The participating families chose the day of the beginning of dietary recording within a given period. When exact weighing was not possible—e.g., in case of eating out—household measures (e.g., spoons, cups) and a photo booklet with foods in toddlers’ portion sizes [39], supplemented with special VG and VN foods, allowed semi-quantitative recording. Besides written information on dietary recording, a video tutorial was provided on the study website. The study staff assessed missing data, requesting the information from the parents via e-mail. Breast milk intakes were estimated by multiplying the reported number of breast meals with age-specific median amounts of breast milk volumes from the DONALD study (Table S1). In this study, breast milk amounts were assessed using test weighing before and after each breast meal [38]. Implausible estimated high breast milk intakes due to highly frequent breastfeeding (12–17 daily feeds, resulting in ≥800 g breast milk/d; *n* = 3, age 1 year) were replaced with the highest non-outlier value. Energy and nutrient intakes were calculated using the food composition database LEBTAB [40]. The composition of staple foods is based on standard German food composition tables BLS 3.02. The energy and nutrient contents of commercial food products, i.e., processed foods and ready-to-eat-meals or snack foods, were estimated by recipe simulation using labeled ingredients and nutrient contents. LEBTAB is continuously updated by adding those products or supplements recorded by study participants.

#### 2.3.2. Anthropometrics

Either the parents or a pediatrician proxy-assessed reported BW and BH during the last medical check-up. If the measurement was older than two weeks before the dietary record, the parents were asked to add the date of assessment. In the case of missing BW and BH (*n* = 2), the age- and sex-specific medians were used.

#### 2.3.3. Covariates

Data on sociodemographic, lifestyle, and early life variables were collected via an online questionnaire using questions partially according to a representative health survey in Germany (German Health Interview and Examination Survey for Children and Adolescents) [41]. The socioeconomic status was assessed using the Winkler Index—a combination of three social status scores (education, profession, total net household income) calculated from parents’ online questionnaire data (1–7 points, each). The higher score of either the mother or the father was used as family socioeconomic status (SES) index and categorized into low (3–8), middle (9–14), or high (15–21) social status according to [42]. Depending on the number of inhabitants, urbanicity was classified into rural (<5,000 inhabitants), small-size urban (5,000–<20,000 inhabitants), medium-size urban (20,000–<100,000 inhabitants), or metropolitan (≥100,000 inhabitants) in accordance with [43]. Physical activity was categorized into active or very active (i.e., “playing outside” and/or “attendance in play/sport groups” ≥4–7 times per week) or less active (<4 times/week). Paternal body mass index (BMI) and BH was used as covariable. Maternal BMI was excluded due to changes in BW postpartum, in particular in breastfeeding women. Small for gestational age (SGA) exact *z*-scores and percentiles of BW(and length) for sex-specific gestational age were assessed via Excel calculator (www.ucalgary.ca/fenton) according to Fenton et al. (2013) [44]. A birth weight ≤10th percentile was defined as SGA, and ≥90th percentile was defined as large for gestational age (LGA). All children between >10th and <90th percentiles were defined as appropriate for gestational age (AGA) [45]. The season of dietary recording was categorized into spring (March–May), summer (June–August), autumn (September–November), and winter (December–February).

#### 2.3.4. Diet Group Classification

Three diet groups (i.e., VG, VN, or OM diet) were categorized according to the following question: “How is your child raised?”

- vegetarian (no meat, sausage, fish, but with dairy products and/or eggs);
- vegan (no meat, sausage, fish, dairy products, or eggs);
- omnivorous (with meat and/or sausage and/or fish).

If parents declared their children to be VG or VN, they were asked whether there are exceptions in food intake, for example VN children drinking cow’s milk or VG children occasionally eating fish. Accordingly, VG and VN children who usually eat meat or fish ≥1 time/week were reclassified as OM (8 VG, 1 VN). VN who usually eat dairy products and/or eggs ≥1 time/week were categorized as VG (24 VN).

#### 2.3.5. Data Analysis and Statistics

Statistical analysis was performed with SPSS Version 20 (IBM SPSS Statistics, Chicago, IL, USA). Total energy and nutrient intake were calculated as individual means of the three recorded days. Dietary energy density (DED) was calculated excluding non-caloric beverages according to [46]. Protein intake was expressed as g/kg BW and fiber intake as g/1,000 kcal. Carbohydrates, added sugar, and fat are presented as percentages of energy intake (%E). Age_Diet was calculated as the difference between the first day of dietary recording and the date of birth. Age_Diet was used to compare the energy and nutrient intake to the German dietary reference values (DRVs). The date of the measurement of BW and BH was used to calculate age at the time of anthropometric assessment (Age_Anthro). BW and BH were analyzed separately for boys and girls, using WHO Anthro version 3.2.2 for SPSS [47]. Severely wasting (weight-for-height *z*-scores <−3 SDs from the WHO standard median), wasting (<−2 SDs), normal weight (>−2 SDs to 2 SDs), overweight (>2 SDs) and obesity (>3 SDs) were classified in accordance with the WHO Growth Standards. Children with height-for-age *z*-scores <−3 SD and <−2 SD from the WHO standard median were classified as severely stunted or stunted, respectively. Weight-for-age *z*-scores <−3 SD and <−2 SD from the median indicate severe underweight and underweight, respectively [48]. Biologically implausible values (*n* = 1) were removed from the analysis according to the WHO recommendations (length/height-for-age ≤−6 or ≥6) [49]. Weight-for-height (wasting) is seen as a short-term indicator for inadequate dietary intake or nutrient utilization that can cause long-term decreases in height-for-age (stunting) [50,51].

Participants’ characteristics are presented as mean ± standard deviation (SD) for the variables with normal distribution or as median and interquartile range (IQR) for the variables that were non-normally distributed. Differences in categorical characteristics between diet groups were tested using a Chi^2^ test or Fisher’s exact test. For continuous characteristics, ANOVA for parametric or Kruskal–Wallis tests for non-parametric data were applied. In the case of significant differences, pairwise Bonferroni post hoc tests (parametric data) or Mann–Whitney U tests (non-parametric data) were used.

Continuous variables were included in the analysis of covariances (ANCOVA) as covariates (children’s age, breastmilk intake, paternal BMI, SES, and weight-for-height *z*-score) and categorical variables as fixed factors (sex, physical activity, and season). In case of unequal categorical variables (e.g., urbanicity), these variables were dummy-coded and included as covariates if necessary. For the analysis of anthropometrics, birth weight (dummy coded) and paternal BH were additionally considered as potential covariates. Total energy intake (TEI) was only considered as a confounder for variables that were not calculated in relation to energy intake. The covariates sex and age were included in the basic model. Each covariate was then checked separately for interaction effects with sex and age. Those covariates or interactions with a *p*-value ≤0.1 and/or a partial eta squared (η^2^) ≥0.06 were added to the model. The backward method was used to build the final model with the same criteria (*p*-value ≤0.1 and/or partial η^2^ ≥0.06). The presented results show the basic models adjusted for age and sex and fully adjusted final models. Due to the large number of tests, *p* ≤0.01 is considered to indicate marginal statistical significance, *p* ≤0.001 indicates statistical significance, and *p* ≤0.0001 high statistical significance to reduce the chance of type I errors. An η^2^ ≥0.01 is interpreted as small, η^2^ ≥0.06 as medium, and η^2^ ≥0.14 as a large effect size [52]. Sensitivity analyses without outliers (modulus of standardized residuals >3) were carried out. The only remarkable differences in the results of significance or effect size are stated in the results.

## 3. Results

### 3.1. Sample Characteristics

Nine hundred and forty-four parents registered their children via online questionnaire; 503 gave their informed consent to participate with their children in the study and met all inclusion criteria. Among these, 131 parents did not complete the 3-day weighted dietary record or the parents’ questionnaire. Another four children were excluded due to diagnosed diseases or dietary therapy. Inclusion of 62 DONALD study participants resulted in a total number of 430 children (127 VG, 139 VN, and 164 OM) (Figure S1).

Sample characteristics are presented in Table 1. The diet groups did not differ with respect to age and sex, with more than 50% of the children being 1–<2 years (Table S2). The majority of families lived in metropolitan or medium-sized urban cities. There were no significant differences in the percentage of children who had ever been breastfed between the diet groups according to the questionnaire. Among the children that had ever been breastfed, on average VN children were breastfed, exclusively and in total, longer than VG and OM children (*p* < 0.001). More VN children (48.6%) were continuously breastfed during the dietary recording than VG (27.1%) or MK (9.9%) children (*p* < 0.0001). However, the average estimated breast milk intake per day was not significantly (n.s.) different between the groups. The median SES was high with no significant differences between the three groups. More than half of the VG and VN parents mentioned ethical motivations as the main motive for choosing their diet and more than 80% started their child’s diet with the introduction of supplementary food (Table S2). Most children were active or very active. More VN children were SGA compared to OM (*p* < 0.01, marginally significant). All seasons of the year were almost equally represented, with slightly more dietary records of VG in autumn and winter and fewer dietary records of VN in summer (n.s.). Median paternal BMI was higher in fathers of OM compared to VG and VN children (*p* < 0.01, marginally significant), with significant differences in paternal BH between fathers of VG and VN children (*p* < 0.001). More VN parents (51.4%) had a high percentage of organic food purchases (≥75% of total food purchases) in comparison to VG (36.0%) and OM parents (12.3%) (*p* < 0.0001, Table S2).

### 3.2. Energy and Nutrient Intakes

TEI and DED did not differ significantly between the diet groups in the final model. After adjustment for confounders, OM children had the highest intake of total protein, total fat, and added sugars. In contrast, VN children had the highest intake of carbohydrates and fiber (all highly significant and of medium to high effect size: *p* ≤ 0.0001, partial η^2^ = 0.06–0.39) (Table 2 and Table S3). By excluding outliers (*n* = 3), there was a significant difference in the added sugar intake between VN and OM children (*p* = 0.001, small effect size partial η^2^ = 0.04).

### 3.3. Anthropometrics

**Figure 1:**
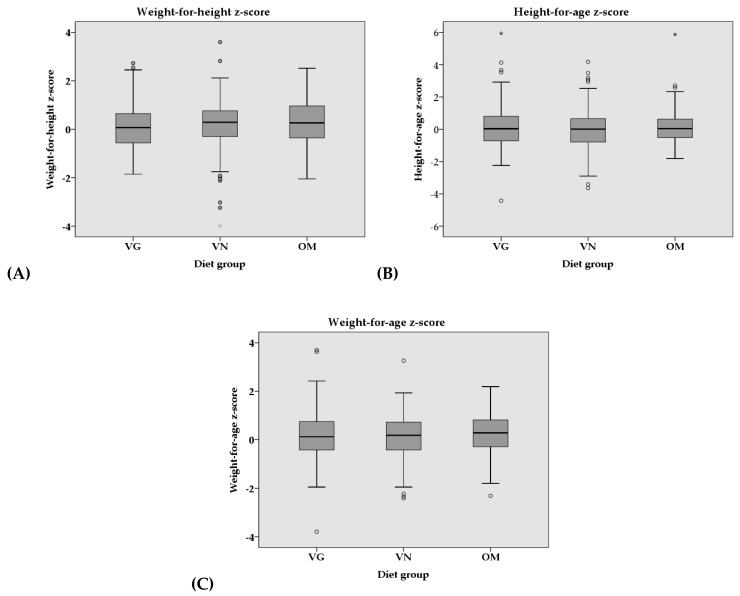
Boxplots of (**A**) weight-for-height, (**B**) height-for-age, and (**C**) weight-for-age *z*-scores of VG, VN, and OM children in the VeChi Diet Study by diet group (127 VG, 139 VN, and 164 OM). VG: vegetarian, VN: vegan, OM: omnivorous. The ° and * represent outliers (1.5-fold and 3-fold IQR, respectively).

**Figure 2:**
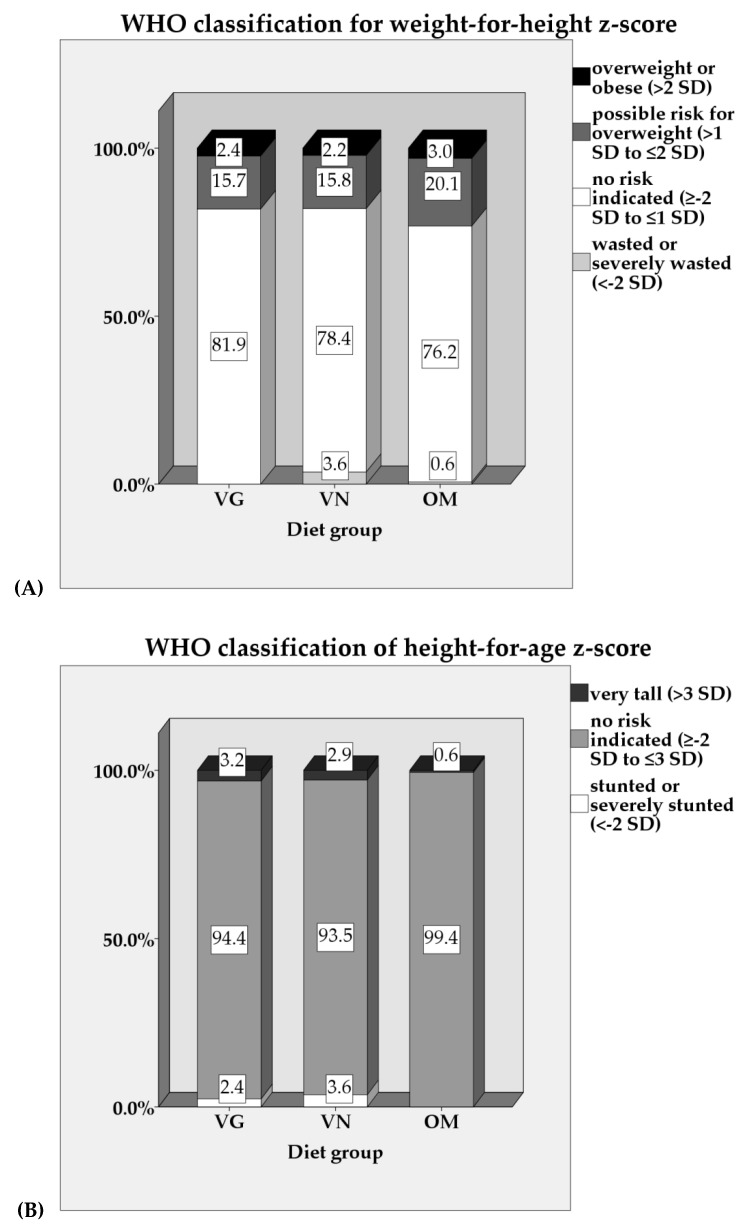
(**A**) Weight-for-height *z*-score and (**B**) height-for-age z-score according to the WHO Growth Standards [49] of VG, VN, and OM children in the VeChi Diet Study by diet group (127 VG, 139 VN, and 164 OM). VG: vegetarian, VN: vegan, OM: omnivorous.

Median weight-for-height, height-for-age, and weight-for-age *z*-scores did not differ significantly between the groups (Figure 1), even after adjustment for various covariates (Table 3 and Table S4). Nevertheless, a slightly higher percentage of VN children (3.6%), compared with VG (2.4%) and OM (0%) children, were classified as stunted according to the WHO child growth standards [49], whereas 3.6% of VN and 0.6% of OM were classified as wasted (VG: 0%). In contrast, a higher percentage of OM children (23.2%) than VG and VN children (18.1% and 18.0%, respectively) were classified as overweight or at possible risk of overweight (Figure 2). Table S5 shows that stunted children had normal weight-for-height (*n* = 4) or were even overweight (*n* = 1) or at possible risk for overweight (*n* = 3). In wasted children, there was no risk indicated, with the exception of one child being classified as very tall.

## 4. Discussion

In the VeChi Diet Study, TEI, DED, and anthropometrics did not differ significantly between VG, VN, and OM young children. However, significant differences in the macronutrient pattern were observed. The intake of total protein, total fat, and added sugars increased (OM > VG > VN) in relation to the degree of inclusion of animal-derived food. On the other hand, the intake of carbohydrates and fiber rose with increasing plant-derived foods (VN > VG > OM).

To our knowledge, there are currently no other studies with VG and VN children at the age of 1–3 years in Western societies. Therefore, we compared our results with the results of studies whose samples at least included children in this age range (e.g., 2–10-year-old children [35]).

There was no difference in TEI between the diet groups, but on average all groups were below the German reference value (for 1–<4-year-old female 1,100 kcal, male 1,200 kcal, according to a physical activity level [PAL] of 1.4 [53]). Besides, the DED did not differ significantly between the groups, but tending to be more energy-dense with OM diets. These results are in accordance with studies from Poland, which also did not find differences in TEI between VG and OM children [31,54], whereas the Polish children additionally met the recommended daily intake on average [29,31,35]. In other studies, the TEIs of VN and VG children were more likely to be lower than those of the OM control groups and/or the reference values [27,34,36]. Our findings that none of the groups met the reference value for TEI on average can be explained by the fact that ≥50% of the children in all groups were younger than 2 years (Table S2). While the DGE does not distinguish between 1-, 2-, or 3-year-old children in their reference values, the European Food Safety Authority (EFSA) does [55]. Compared to the more precise EFSA reference values, the average TEIs of all our diet and age groups were higher than this reference (Table S6). Another approach is to calculate the individual energy requirement with the equations published by Henry (2005) for 0 to 3-year-old males and females (m: BMR = (28.2 × BW) + (859 × (BH/100)) − 371; f: BMR = (30.4 × BW) + (703 × (BH/100)) – 287 [56]), multiply the BMR by a PAL of 1.4, and add 10% for growth in accordance with the approach of the DGE [6]. Using this approach, the estimated energy requirement was lower than the German dietary reference value (1,004 ± 200 kcal) and closer to the mean TEI of the study groups. However, 30.8% of VG, 27.8% of VN, and 41.5% of OM children did not meet their calculated individual energy needs. Nevertheless, to assess whether the individual TEI is inadequate, anthropometrics of the children should always be taken into account [57].

On average, all groups had 2.3–2.5-fold higher protein intakes than the German reference value (1 g protein/kg BW and day), with OM children having the highest average intake. This concurs with findings from Thane and Bates (2000), where British OM children (*n* = 1,307, 1.5–4.5 years) showed a significantly higher percentage contribution to energy from protein compared to VG children (*n* = 44, 1.5–4.5 years), but both groups had >2-fold higher intakes than the reference nutrient intake [36]. Sanders (1988) found that all VN children (39 VN children, 1–7 years, UK) in their study reached at least the minimum of 10%E from protein [27], whereas the average protein intake among Polish VG children (11.9–16.0%E, 5–11/2–10/2–18 years, including VN) did not differ significantly from the OM control groups (14.2–16.0%E, 5–11/2–10/2–18 years) [28,29,31,54]. Some experts propose a higher protein intake for VG and VN children due to the lower digestibility and/or protein quality of plant protein. In the VeChi Diet Study, VG and VN children met these higher recommendations on average (<2 years: 30–35%, 1.6–1.7 g/kg BW per day; 2–6 years: 20–30%, 1.4–1.6 g/kg BW per day [58]). In our study, the maximum individual protein intake reached 18.6%E (VG 15.9%E, VN 16.3%E, OM 18.6%E), which is within the US acceptable macronutrient distribution range (AMDR) for protein (10–35%E) [59] and in accordance with other studies on OM infants and toddlers [60]. An excess protein intake during early childhood is thought to cause adverse effects with respect to kidney function and development of overweight, but protein source—i.e., plant, meat, or dairy protein—has to be considered. In adults, long-term consumption of red meat may increase the risk for chronic kidney diseases, whereas white meat and dairy protein seem to have no effect, and plant protein seems to be renal protective [61]. However, the evidence of the current literature is limited and does not allow us to draw a final conclusion [61,62]. Additionally, a high protein intake (≥4 g protein/kg BW per day) during the first 2 years of life is thought to be associated with a higher risk for overweight or obesity later in life [60,63]. Therefore, some experts suggest setting a maximum acceptable level of 14%E from protein for 12–24-month-old infants [64], in particular by limiting the intake of unmodified cow milk during the second year of life [60].

The fat intake only differed significantly between VN and OM children after adjusting for covariates in the final model, with OM children having a higher adjusted fat intake. On average, all diet groups met the German reference for fat intake of 30–40%E [53]. This result is consistent with the findings of the aforementioned British study by Thane and Bates (2000), where the fat intake of VG children tended to be lower than that of OM children, and all diet groups had average fat intakes from 33.6 to 36.3%E [36]. In a Polish study by Laskowska-Klita et al. (2011), VG children did not meet the recommended daily fat intake on average (27.5% ± 6.9%E) [29]. In another study with VN children in the UK, the fat intake was on average 30%E (16–39%E) [27]. However, besides fat quantity, fat quality is (maybe even more) relevant for health [65].

There were no differences in carbohydrate intakes between the groups in the unadjusted analysis. After adjustment for age, sex, breastmilk intake, and urbanicity, VN and OM children varied in a highly significant manner in the final model, with VN children having the highest intakes (Table S3). All groups met the reference intake of ≥50%E on average [53]. These results are in line with the above-mentioned Polish studies that also did not reveal differences between VG and OM children’s unadjusted carbohydrate intakes [28,31,35,54]. In contrast, Thane and Bates (2000) found, also without adjusting for covariates, higher carbohydrate intakes in VG in comparison to OM children (significant only in 3–4.5-year-olds). In all these studies, VG and OM children had carbohydrate intakes above 50%E [28,29,31,35,36]. Nevertheless, in recent years, the carbohydrate quantity has been considered less relevant for health than the quality [66] characterized by the glycemic index, whole grain intake, added sugar, and fiber intake.

On average, OM children had higher unadjusted intakes of added sugars in comparison to VN children, with VN children having approximately half the intake of the other two groups. After adjustment for age, sex, breastmilk intake, SES, paternal BMI, and seasons, the differences were no longer significant—with the exception of the difference between VN and OM children when outliers were excluded. The median added sugar intake (unadjusted) of all groups was below the 5 and 10%E WHO limit for free sugar intake [67]. In the British study by Thane and Bates (2000), there were also no significant differences in the (total) sugar intake of VG compared to OM children [36].

Instead, there were highly significant differences between all groups in the fiber intake per 1,000 kcal, with VN children having the highest average intake, followed by VG and finally OM children. This confirms the results of other studies, where VG had higher fiber intakes than OM children [28], although these differences were sometimes not significant [36]. Up to now, there is no German reference intake for dietary fiber for toddlers, but the value of 10 g/1,000 kcal is considered to be attainable [6], which all diet groups in the VeChi Diet Study exceeded. Some VG and VN children of the VeChi Diet Study (VG: 3, VN: 16) even had very high intakes of fiber (30–45 g/day). The Scientific Society for Vegetarian Nutrition (SSVN) recommends limiting fiber intake in early childhood in VN children because in a high-fiber diet, the calorie density of meals is decreased due to the increase in total food volume, and the absorption of protein, fat, and minerals could be impaired [68]. Therefore, if the growth of VN or VG children is inappropriate, a decrease in fiber intake should be considered.

In the VeChi Diet Study, anthropometrics did not significantly differ between the diet groups and indicated on average normal growth in all groups. However, more VN and VG than OM children were classified as stunted or wasted. For interpreting these results, it has to be considered that the WHO Growth Standards describe “how children should grow when not only free of disease but also when reared following healthy practices such as breastfeeding and a non-smoking environment.” These standards “can be used to assess children everywhere, regardless of ethnicity, socioeconomic status and type of feeding” [69]. Therefore, despite the cross-sectional design of our study, the deviations observed in those children who were classified as stunted or wasted represent abnormal growth. Stunting reflects long-term inadequate dietary intake that could result, amongst other reasons, from an unbalanced VG or VN diet, with a low DED, a low protein or zinc intake, or multiple nutritional deficiencies that interact with other adverse environmental factors (e.g., infections) [51,70].

Regarding these eight children classified as stunted, two had very low reported energy intakes (534 kcal/day and 598 kcal/day, respectively), and both were exclusively breastfed >6 months (7 and 9 months, respectively). An overly long period of exclusively breastfeeding can result in an insufficient intake of complementary foods and inadequate low TEI because, after a certain age, human milk alone cannot supply energy and all nutrients in adequate amounts to meet a child’s requirements [71]. Furthermore, one of the two children as well as three other children classified as stunted had parents with a BH (mother: 161 cm, father: 170 cm) below the German average (167 cm and 180–181 cm of 25–55-year-old women or men, respectively) that might have influenced the child’s BH. The other child with low energy intake was also categorized as SGA, which is considered a risk factor for stunting [72]. Another stunted child was categorized as SGA, and its birthweight was only slightly above 2500 g (2545 g). The seventh child was exclusively breastfed for twelve months (the eighth child was breastfed for eight months), and it had parents with BHs (mother: 160 cm, father: 178 cm) below the German average. None of the children had been diagnosed with chronic diseases, but we do not have any information of serial infections or inflammations that could have caused the stunting. Other risk factors for stunting were not observed, e.g., smoking during pregnancy or lactation. Protein intake was adequate in all children. Further nutrients, which have been associated with growth, i.e., zinc or iron, have not yet been analyzed.

With the exception of the aforementioned eight children, our findings assume a normal child development indicated by average anthropometrics in the normal range. This is in line with other studies among 1–3-year-old VG and VN children, indicating a tendency to be smaller and lighter—slightly below the 50th percentile of the reference—in comparison to standards [14,27,30,34] and/or no differences on average in comparison to OM children [28,31,33,54,73]. O’Connell et al. (1989) found significant differences between the mean z-scores of height-for-age between VG children (of which 83% were VN) and the US reference population, but only for children ≤5 years. Overall, 8% of the VG children in that study had heights-for-age, 3% weights-for-age, and 1% weights-for-height that were <5th percentile of the reference. On average, weight-for-height was slightly higher than those of the reference population (significant different only ≤5 years and at age 9 years) [14]. This could be at least in part explained by some irregularities within the reference population, e.g., being formula-fed instead of breastfed [68]. However, the use of the WHO Growth Standards results in a higher prevalence of stunting and wasting than the formerly used WHO/National Center for Health Statistics reference [74,75]. As a result, the prevalence of stunted and wasted children observed in the VeChi Diet Study might have been even higher than the prevalence in O’Connell et al. (1989).

Studies with macrobiotic and non-macrobiotic VG children in the US showed anthropometric values in the reference limits [76] or slightly below [72,77,78]. In the Netherlands, macrobiotic VN children exhibited retarded development [79,80,81,82]. Due to the fact that a macrobiotic diet, especially practiced in the 1970s and 1980s, obviously differs remarkably from a current VG or VN diet, these groups and their diets are not comparable to the children in our study.

On the other hand, slightly more OM (3.0%) than VG and VN children (2.4% and 2.2%, respectively) were classified as overweight. This is less than in a recent large study in Germany (7.2–8.0% overweight and 3.3–4.6% obese at the age of 1–3 years [83]). VG and VN diets are discussed to be protective against childhood obesity [18,20]. The low prevalence and the marginal difference between the diet groups in our study might be due to the high SES compared to the general German population.

Some strengths and limitation of our study have to be discussed. One major limitation of this study is the proxy-reported BW and BH (by parents or pediatrician). Hence, these data are more vulnerable for bias [84,85]. Furthermore, the cross-sectional design allows for only a glance at food intake and anthropometrics. Nevertheless, follow-up investigations with further examinations (measured anthropometrics, nutrient status in blood and urine) are planned in order to assess the long-term development of VG and VN compared to OM children. The inclusion of children in the DONALD study resulted in an overrepresentation of participants living in the federal state North Rhine-Westphalia (33.7% of all study participants) where the DONALD study is located. Another limitation is the estimation of breastmilk intake, which is based on reliable data of the DONALD study but is not as exact as weighing the children before and after each breastfeeding. Additionally, a 3-day period of dietary recording has been said to be insufficient to estimate the habitual energy and nutrient intake [86]. Nevertheless, only two days of recording were required for assessing micronutrients such as iron, magnesium, zinc, or ascorbic acid [87]. To increase compliance and not to overburden the parents of very young children, three days of dietary records were considered. Additionally, the classification into the diet groups could be criticized. It is well known from adult studies that measured food intake did not always agree with self-characterization of diet groups [88,89,90,91]. Therefore, in the VeChi Diet Study, we mainly focused on parent-reported categorization, which we corrected if animal-derived foods were consumed ≥ 1 time/week. This is because a maximum of consuming animal foods three times per month probably does not affect nutrient intake and status, and compromises are being made in the implementation of a vegan diet in everyday life.

A major strength is the large sample within a narrowly defined age group. Due to differences in growth rate and development, and the diverse needs in different stages of childhood, preschool children should not be considered in the same study groups as, e.g., adolescents. Another strength is the relative balance of the study groups with approximately one-third of the participants in each diet group, with only slightly more female than male children and no significant differences in age, urbanicity, SES, estimated physical activity, and birth weight categories. Moreover, we used weighed dietary records because they provide the best estimate for children aged 0.5–4 years [92]. The prospective survey did not depend on the parents’ ability to recall the food intake of their children. Parents were instructed to maintain the usual diet, and every protocol was checked for completeness and plausibility. Missing information was immediately collected from parents. As seen in other investigations, underreporting is unlikely (only 1%) in 1–5-year-old children [93]. Furthermore, as VG and VN diets tend to include special foods—e.g., meat substitutes, milk alternatives, special dietary supplements such as protein powder, and fortified products—such a detailed method is essential for dietary surveys in these diet groups. The nutrient database LEBTAB ensures a high accuracy in nutrient intake due to brand-specific estimations of ingredients and nutrient contents by recipe simulation including fortification. The survey period covered all seasons of the year with no significant differences between the study groups, and parents were asked to include weekdays as well as weekends in the record.

## 5. Conclusions

In conclusion, our results indicate that a VG and VN diet in early childhood provides comparable amounts of energy and a macronutrient pattern in accordance with recommendations and can ensure normal growth, as there were no significant differences in proxy-reported anthropometrics compared to OM children of the same age. However, the observed small percentage of VG and VN children in our sample classified as stunted should emphasize the importance of adequate energy and nutrient intake for children on VG and VN diets. Finally, the population of the VeChi Diet Study provides a suitable baseline cohort for future long-term investigations studying the effects of VG and VN diets during childhood, adolescence, and on into adulthood.

## Figures and Tables

**Table 1:** Sample characteristics of the VeChi Diet Study by diet group.

	VG	VN	OM
Total ^3^	127 (29.5)	139 (32.3)	164 (38.1)
**Sex ^3 x^**			
Female	64 (50.4)	76 (54.7)	83 (50.3)
Male	63 (49.6)	63 (45.3)	81 (49.4)
**Age ^2 z^**			
Age_Diet (year)	2.0 (1.4)	1.8 (1.3)	2.0 (1.5)
Age_Anthro (year)	1.9 (1.4)	1.8 (1.4)	2.0 (1.5)
**Child weight and height ^1 z^**			
BW (kg)	12.1 ± 2.3	12.0 ± 2.5	12.7 ± 2.6
BH (cm)	86.6 ± 8.8	85.6 ± 8.8	88.2 ± 9.3
**Urbanicity ^3 x #^**			
Rural (<5,000)	19 (15.0)	28 (20.1)	28 (17.2)
Small-size urban (5,000-<20,000)	16 (12.6)	20 (14.4)	17 (10.4)
Medium-size urban (20,000-<100,000)	33 (26.0)	31 (22.3)	31 (19.0)
Metropolitan (≥100,000)	59 (46.5)	60 (43.2)	87 (53.4)
**Breastfeeding**			
Ever breastfed ^3 y^	121 (95.3)	138 (99.3)	157 (95.7)
Duration of exclusively breastfeeding (mo) ^2 z^	5.0 (2.0) ^a^	6.0 (2.0) ^a,b^	5.0 (2.0) ^b^
Duration of total breastfeeding (mo) ^2 z^	13.1 (10.0) ^a^	15.9 (10.0) ^a,b^	11.1 (7.0) ^b^
Breast milk intake in dietary record (if breastfed) (g/day) (*n* = 119) ^2 z^	275.0 (186.3)	303.3 (280.0)	163.3 (316.7)
**SES (Winkler Index score D2004) ^3 y^**			
Low (3–8)	3 (2.4)	2 (1.4)	0 (0)
Middle (9–14)	31 (24.4)	37 (26.6)	30 (18.3)
High (15–21)	93 (73.2)	100 (71.9)	134 (81.7)
**Physical activity ^3 x ##^**			
Less active (<4 times/week)	71 (55.5)	70 (50.0)	64 (39.8)
Active or very active	57 (44.5)	70 (50.0)	97 (60.2)
**Birth weight categories ^3 x #^**			
Small for gestational age (SGA)	23 (17.8)	26 (18.7)	25 (15.5)
Appropriate for gestational age (AGA)	101 (78.3)	99 (71.2)	134 (83.2)
Large for gestational age (LGA)	5 (3.9)	14 (10.1)	2 (1.2)
**Season of dietary record ^3 x^**			
Spring	21 (16.5)	45 (32.4)	36 (22.0)
Summer	20 (15.7)	17 (12.2)	38 (23.2)
Autumn	41 (32.3)	36 (25.9)	43 (26.2)
Winter	45 (35.4)	41 (29.5)	47 (28.7)
**Paternal BMI and height**			
BMI (kg/m^2^) ^2 z ##^	24.5 (3.9)	24.3 (4.1)	25.7 (3.1)
BH (cm) ^1 z ##^	183 ± 7 ^a^	180 ± 7 ^a^	182 ± 7

Values are ^1^ mean ± standard deviation (SD), ^2^ median and (interquartile range, IQR), ^3^ frequencies (percentage); VG: vegetarian, VN: vegan, OM: omnivorous, BW: body weight, BH: body height, Age_Diet: age at dietary record, Age_Anthro: age at anthropometric measurement, BMI: body mass index. Differences were analyzed using ^x^ Chi^2^-test, ^y^ Fisher’s exact test for cell frequencies of <20% of expected count less than 5, ^z^ ANOVA and Bonferroni post hoc tests for parametric, Kruskal–Wallis test and Mann–Whitney U test for nonparametric, continuous data. ^a,b^ exponents indicate statistical significance (at least *p* ≤ 0.001). ^#^ missing *n* = 1, ^##^ missing *n* = 5.

**Table 2:** Average daily intake of energy and macronutrients of vegetarian (VG), vegan (VN), and omnivorous (OM) children in the VeChi Diet Study by diet group.

	Median (IQR)			Basic model (age, sex adjusted)		Final model	
	VG (*n* = 127)	VN (*n* = 139)	OM (*n* = 164)	*p*-value	Partial η^2^	*p*-value	Partial η^2^
**TEI ^a^**, kcal/day	956 (790–1084)	986 (821–1186)	974 (856–1099)	0.281	0.006	0.055	0.015
**DED ^b^**, kcal/g	1.12 (0.98–1.33)	1.05 (0.93–1.22)	1.15 (1.02–1.35)	0.009 ^#^	0.022	0.466	0.004
**Protein ^c^**, g/kg BW	2.26 (1.83–2.65) ^1^	2.25 (1.82–2.76) ^2^	2.54 (2.16–3.06) ^1,2^	<0.0001 ***	0.054	<0.0001 ***	0.122
**Fat ^d^**, %E	33.7 (29.7–36.6)	33.6 (27.9–39.4) ^1^	32.6 (28.2–37.2) ^1^	0.781	0.001	<0.0001 ***	0.049
**Carbohydrates ^e^**, %E	53.6 (50.5–58.2)	53.8 (49.4–59.3) ^1^	53.1 (47.9–57.1) ^1^	0.029	0.017	<0.0001 ***	0.070
**Added sugars ^f^**, %E	4.2 (1.1–6.6)	2.1 (0.6–5.7)	4.8 (2.2–8.7)	<0.0001 ***	0.045	0.002 * ^##^	0.032
**Fiber ^g^**, g/1,000 kcal	16.1 (13.8–20.0) ^1^	19.6 (16.3–24.1) ^1^	13.4 (10.1–16.6) ^1^	<0.0001 ***	0.231	<0.0001 ***	0.290

Values are unadjusted median (IQR), whereas *p*-values and effect sizes were derived from ANCOVA (Table S3) and adjusted for age and sex (basic model) or other confounders (final model, see below). Sensitivity analyses without outliers (|standardized residuals|>3) were carried out. No remarkable differences in the results of significance or effect size were found (if not stated otherwise). VG: vegetarian, VN: vegan, OM: omnivorous, BW: body weight, TEI: total energy intake, DED: dietary energy density, %E: % of TEI, SES: socioeconomic status. * *p* ≤ 0.01 marginal statistical significance, *** *p* ≤ 0.0001 high statistical significance, Bonferroni adjusted. ^#^ marginal significance disappears without outliers (|standardized residuals|> 3), *p* = 0.013, partial η^2^ = 0.021. ^##^ statistical significance between VN and OM children without outliers (|standardized residuals|>3), *p* = 0.001, partial η^2^ = 0.035. ^1,2^ exponents indicate statistical significance in the final model (at least *p* ≤ 0.001). ^a^ Final model adjusted for age, sex, breastmilk intake, SES, and seasons (*n* = 430). ^b^ Final model adjusted for age, sex, breastmilk intake, SES, paternal BMI, and seasons (*n* = 425). ^c^ Final model adjusted for age, sex, breastmilk intake, SES, weight-for-height *z*-score, TEI, paternal BMI, and seasons (*n* = 425). ^d^ Final model adjusted for age, sex, breastmilk intake, and urbanicity (*n* = 429). ^e^ Final model adjusted for age, sex, breastmilk intake, and urbanicity (*n* = 424). ^f^ Final model adjusted for age, sex, breastmilk intake, SES, physical activity, weight-for-height *z*-score, paternal BMI, and seasons (*n* = 421). ^g^ Final model adjusted for age, sex, breastmilk intake, SES, weight-for-height *z*-score, and urbanicity (*n* = 429).

**Table 3:** Average weight-for-height, height-for-age, and weight-for-age *z*-score of VG, VN, and OM children in the VeChi Diet Study by diet group (127 VG, 139 VN, and 164 OM).

	$\bar{x}$ ± SD			Basic Model (age, sex adjusted)		Final Model	
*z*-Score	VG (*n* = 127)	VN (*n* = 139)	OM (*n* = 164)	*p*-value	Partial η^2^	*p*-value	Partial η^2^
**Weight-for-Height ^a^**	0.11 ± 0.95	0.16 ± 1.08	0.23 ± 0.96	0.540	0.003	0.488	0.004
**Height-for-Age ^b^**	0.11 ± 1.34	0.01 ± 1.26	0.13 ± 1.01	0.569	0.003	0.055 ^#^	0.016
**Weight-for-Age ^c^**	0.17 ± 0.99	0.11 ± 0.93	0.25 ± 0.87	0.344	0.005	0.061	0.014

Values are unadjusted arithmetic mean ± standard deviation (SD), whereas *p*-values and effect sizes were derived from ANCOVA (Table S4) and adjusted for age and sex (basic model) or other confounders (final model, see below). Sensitivity analyses without outliers (|standardized residuals|>3) were carried out. No remarkable differences in the results were found (if not stated otherwise). VG: vegetarian, VN: vegan, OM: omnivorous, SES: socioeconomic status. Bonferroni adjusted. ^#^ marginal significance appears without outliers (|standardized residuals |>3), *p* = 0.007, partial η^2^ = 0.027 (VG vs VN, *p* = 0.005). ^a^ Final model adjusted for age, sex, physical activity, SGA, SES, paternal BMI, and seasons (*n* = 423). ^b^ Final model adjusted for age, sex, physical activity, SGA, breastmilk intake, TEI, SES, paternal height, urbanicity, and seasons (*n* = 421). ^c^ Final model adjusted for age, sex, physical activity, SGA, breastmilk intake, TEI, and paternal height (*n* = 423).

## Supplementary Materials

The following figures and tables are available online at https://www.mdpi.com/2072-6643/11/4/832/s1. Figure S1: Flow chart of recruitment of vegetarian (VG), vegan (VN), and omnivorous (OM) children in the VeChi Diet Study. Table S1: Median breast milk volumes per meal. Table S2: Further sample characteristics of VG, VN, and OM children in the VeChi Diet Study by diet group. Table S3: Average intake of energy and macronutrients of VG, VN, and OM children in the VeChi Diet Study by diet group. Table S4: Average weight-for-height, height-for-age and weight-for-age *z*-score of VG, VN, and OM children in the VeChi Diet Study by diet group. Table S5: Cross tab of stunted and wasted children of VG, VN, and OM children in the VeChi Diet Study. Table S6: Median energy intake categorized by age group of VG, VN, and OM children in the VeChi Diet Study.
