# Supplementary material for: Energy, Macronutrient Intake, and Anthropometrics of Vegetarian, Vegan, and Omnivorous Children (1–3 Years) in Germany (VeChi Diet Study)
Source: Nutrients. 2019 Apr 12;11(4):832. doi: 10.3390/nu11040832 (PMC6521189; doi:10.3390/nu11040832)
Supplement: Supplementary file 1 [file nutrients-11-00832-s001.pdf]

Table S1. Median breast milk volumes per meal[1].

Age in years	N participants	N meals	Median g/meal (P10-P90)
≥ 0.75 - < 1.0	352	3427	80 (30-170)
≥ 1.0 - < 1.5	160	1327	70 (25-150)
≥ 1.5 - < 2.0	54	419	50 (25-140)
≥ 2.0 - < 3.0	15	97	50 (15-120)
≥ 3.0	3	18	40 (10-40)

[1]Data from the DONALD study of 584 participants and 5,288 meals using a baby scale (Soehnle Multina 8,300) to weight their child before and after feeding to the nearest 10 g [1].

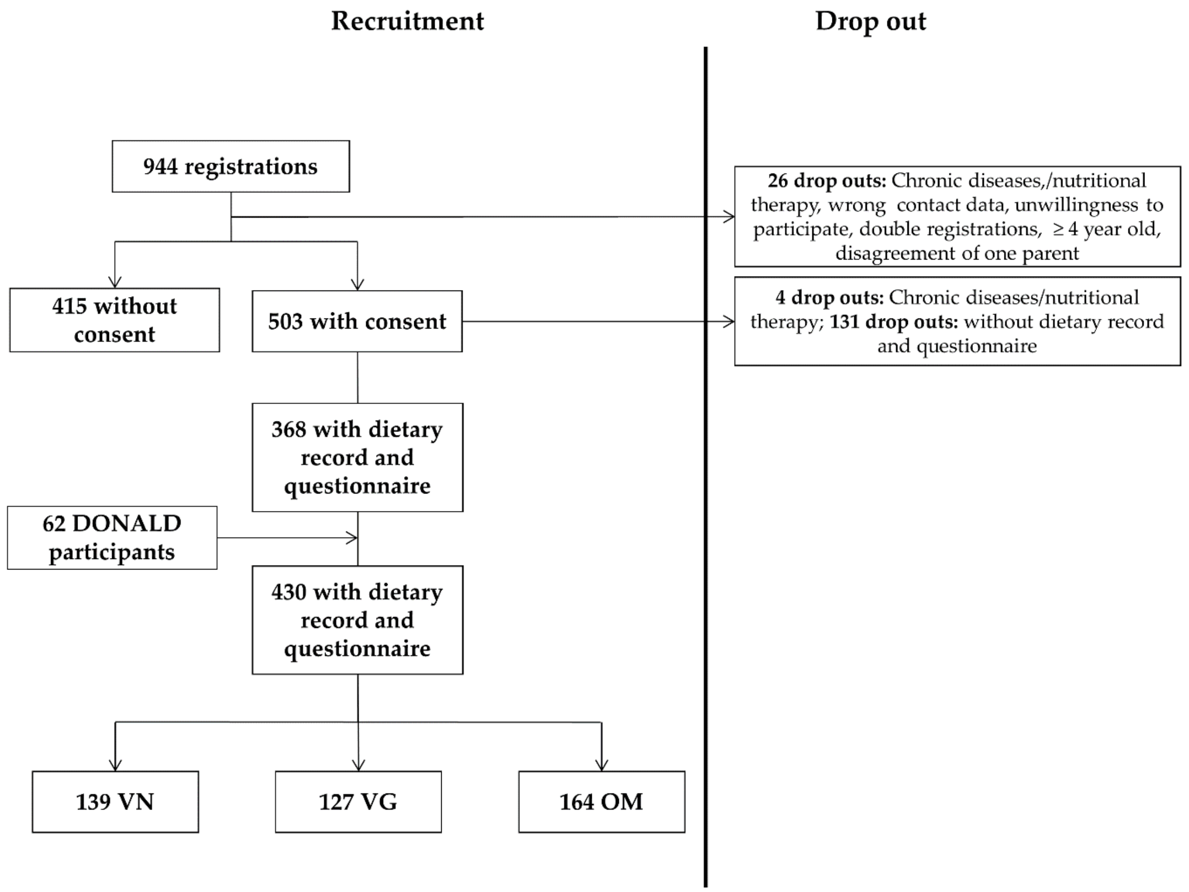

Figure S1. Flow chart of recruitment of vegetarian (VG), vegan (VN), and omnivorous (OM) children in the VeChi Diet Study.

Table S2. Further sample characteristics of VG, VN, and OM children in the VeChi Diet Study by diet group.

	VG	VN	OM
**Total**	**127 (29.5)**	**139 (32.3)**	**164 (38.1)**
**Age group** x			
<2 years	67 (52.8)	81 (58.3)	85 (51.8)
≥2-<3 years	37 (29.1)	38 (27.3)	44 (26.8)
≥3 years	23 (18.1)	20 (14.4)	35 (21.3)
**Main motivation** y #			
Ethical	81 (64.3)	94 (67.6)	-
Health-related	31 (24.6)	37 (26.6)	-
Ecological	6 (4.8)	6 (4.3)	-
Other (social, religious, disgust)	8 (6.3)	2 (1.4)	-
**Start of the diet** x ##			
with the introduction of supplementary food	107 (84.3)	123 (88.5)	140 (85.4)
later	18 (14.2)	16 (11.5)	24 (14.6)
**Organic food purchases** x ###			
Never or <25% of total food purchases	7 (5.6)[1]	14 (10.1)[2]	46 (28.2)[1,2]
≥25-≤50% of total food purchases	29 (23.2)[1]	22 (15.8)[2]	64 (39.3)[1,2]
>50-≤75% of total food purchases	44 (35.2)[1]	32 (23.0)[2]	33 (20.2)[1,2]
>75% of total food purchases	45 (36.0)[1]	71 (51.1)[2]	20 (12.3)[1,2]

Values are frequencies (percentage); VG: vegetarian, VN: vegan, OM: omnivorous. Differences were analyzed using [x] Chi^2-test, [y] Fisher's exact test for cell frequencies of <20% of expected count less than 5. [1,2,3]exponents indicate statistical significance (at least p≤0.001). [#]no query with OM parents, [##]missing n = 2, [###]missing n = 3.

Table S3. Average intake of energy and macronutrients of VG, VN, and OM children in the VeChi Diet Study by diet group.

	Basic model (age, sex adjusted)					Final model				
	**VG**	**VN**	**OM**	**p-value**	**Partial η^2**	**VG**	**VN**	**OM**	**p-value**	**Partial η^2**
**TEI**[a] kcal/d	974.9 (937.9-1011.9)	1014.3 (978.8-1049.7)	984.7 (952.0-1017.3)	0.281	0.006	990.3 (951.0-1029.5)	1053.3 (1010.5-1096.1)	992.5 (955.6-1029.4)	0.055	0.015
**DED**[b] kcal/g	1.16 (1.12-1.21)	1.09 (1.05-1.13)	1.18 (1.14-1.21)	0.009[#]	0.022	1.16 (1.12-1.20)	1.12 (1.08-1.17)	1.14 (1.10-1.18)	0.466	0.004
**Protein**[c] g/kg BW	2.29 (2.17-2.41)[1]	2.37 (2.25-2.49)[2]	2.67 (2.56-2.78)[1,2]	<0.0001***	0.054	2.34 (2.27-2.42)[1]	2.41 (2.34-2.49)[2]	2.69 (2.62-2.75)[1,2]	<0.0001***	0.122
**Fat**[d] %E	33.6 (32.5-34.7)	33.3 (32.2-34.4)	33.1 (32.1-34.1)	0.781	0.001	33.5 (32.1-34.9)	31.2 (30.1-32.4)[1]	36.0 (34.2-37.7)[1]	<0.0001***	0.049
**Carbohydrates**[e] %E	54.0 (52.9-55.1)	54.4 (53.3-55.4)	52.5 (51.6-53.5)	0.029	0.017	54.1 (52.7-55.6)	56.2 (55.0-57.4)[1]	50.1 (48.3-519)[1]	<0.0001***	0.070
**Added sugars**[f] %E	4.4 (3.9-5.0)	3.7 (3.2-4.3))[1]	5.4 (4.9-6.0))[1]	<0.0001***	0.045	4.5 (3.9-5.1)	3.8 (3.2-4.4)	5.3 (4.7-5.8)	0.002* [##]	0.032
**Fiber**[g] g/1.000 kcal	16.7 (15.8-17.6)[1]	20.1 (19.2-20.9)[1]	13.6 (12.8-14.4)[1]	<0.0001***	0.231	16.5 (15.5-17.5)[1]	21.8 (20.9-22.6)[1]	12.2 (10.9-13.5)[1]	<0.0001***	0.290

Values are estimated marginal means and 95% CI for typical cases. Sensitivity analyses without outliers (|standardized residuals|>3) were carried out. No remarkable differences in the results of significance or effect size were found (if not stated otherwise). VG: vegetarian, VN: vegan, OM: omnivorous, BW: body weight, TEI: total energy intake, DED: dietary energy density, %E: % of TEI, SES: socioeconomic status. *p≤0.01 marginal statistical significance, **p≤0.001 statistical significance, ***p≤0.0001 high statistical significance, Bonferroni adjusted. [#]marginal significance disappears without outliers (|standardized residuals|> 3), p = 0.013, partial η^2 = 0.021. [##]statistical significance between VN and OM children without outliers (|standardized residuals|> 3), p = 0.001, partial η^2 = 0.035. [1,2,3]exponents indicate statistical significance in the final model (at least p≤0.001). [a]Final model adjusted for age, sex, breastmilk intake, SES, seasons (n = 430). [b]Final model adjusted for age, sex, breastmilk intake, SES, paternal BMI, seasons (n = 425); [c]Final model adjusted for age, sex, breastmilk intake, SES, weight-for-height z-score, TEI, paternal BMI, seasons (n = 425); [d]Final model adjusted for age, sex, breastmilk intake, urbanicity (n = 429); [e]Final model adjusted for age, sex, breastmilk intake, urbanicity (n = 424); [f]Final model adjusted for age, sex, breastmilk intake, SES, physical activity, weight-for-height z-score, paternal BMI, seasons (n = 421); [g]Final model adjusted for age, sex, breastmilk intake, SES, weight-for-height z-score, urbanicity (n = 429).

Table S4. Average weight-for-height, height-for-age and weight-for-age z-score of VG, VN, and OM children in the VeChi Diet Study by diet group.

	Basic model (age, sex adjusted)					Final model				
**z-score**	**VG**	**VN**	**OM**	**p-value**	**Partial η²**	**VG**	**VN**	**OM**	**p-value**	**Partial η²**
Weight-for-height[a]	0.11 (-0.7-0.28)	0.15 (-0.02-0.32)	0.23 (0.08-0.39)	0.540	0.003	0.07 (-0.10-0.25)	0.18 (0.01-0.35)	0.22 (0.06-0.38)	0.448	0.004
Height-for-age[b]	0.11 (-0.09-0.32)	-0.01 (-0.21-0.19)	0.13 (-0.06-0.31)	0.569	0.003	0.25 (-0.04-0.55)	-0.21 (-0.44-0.02)	0.03 (-0.30-0.35)	0.055[#]	0.016
Weight-for-age[c]	0.16 (-0.00-0.33)	0.10 (-0.06-0.26)	0.26 (0.11-0.40)	0.344	0.005	0.11 (-0.05-0.26)	0.06 (-0.10-0.22)	0.31 (0.16-0.46)	0.061	0.014

Values are estimated marginal means and 95% CI for typical cases. Sensitivity analyses without outliers (|standardized residuals |>3) were carried out. No remarkable differences in the results were found (if not stated otherwise). VG: vegetarian, VN: vegan, OM: omnivorous, SES: socioeconomic status. *p≤0.01 marginal statistical significance, **p≤0.001 statistical significance, ***p≤0.0001 high statistical significance, Bonferroni adjusted. [#]marginal significance appears without outliers (|standardized residuals|>3), p = 0.007*, partial η^2 = 0.027 (VG vs VN, p = 0.005). [a]Final model adjusted for age, sex, physical activity, SGA, SES, paternal BMI, seasons (n = 423); [b]Final model adjusted for age, sex, physical activity, SGA, breastmilk intake, TEI, SES, paternal height, urbanicity, seasons (n = 421); [c]Final model adjusted for age, sex, physical activity, SGA, breastmilk intake, TEI, paternal height (n = 423).

Table S5. Cross tab of stunted and wasted children of VG, VN, and OM children in the VeChi Diet Study.

**Height-for-age** / **Waste-for-Height**	Very tall (>3 SD)	Stunted or severely stunted (<-2 SD)	No risk indicated (≥-2 SD to ≤3 SD)
Overweight or obese (>2 SD)	0	1	0
Possible risk for overweight (>1 SD to ≤2 SD)	0	3	0
Wasted or severely wasted (<-2 SD)	1	0	5
No risk indicated (≥-2 SD to ≤1 SD)	0	4	0

SD: standard deviation, VG: vegetarian, VN: vegan, OM: omnivorous.

Table S6. Median energy intake categorized by age group of VG, VN, and OM children in the VeChi Diet Study.

	**VG**	**VN**	**OM**	**EFSA reference value, PAL = 1.4 [2]**
**Total**	**127 (29.5)**	**139 (32.3)**	**164 (38.1)**	
**Energy intake (kcal/d)**				
<2 years	831 (737-995)	906 (737-1054)	898 (819-1039)	f: 693-717, m: 764-788
≥2-< 3 years	1032 (923-1094)	1040 (932-1201)	1019 (918-1084)	f: 931-955, m: 1003-1027
≥3 years	1084 (935-1264)	1159 (1054-1363)	1105 (983-1275)	f: 1075-1099, m: 1170

Values are medians (IQR). F. female, m: male, VG: vegetarian, VN: vegan, OM: omnivorous.

References

1. Kroke, A.; Manz, F.; Kersting, M.; Remer, T.; Sichert-Hellert, W.; Alexy, U.; Lentze, M.J. The DONALD Study. History, current status and future perspectives. *Eur J Nutr* **2004**, *43*, 45–54, doi:10.1007/s00394-004-0445-7.
2. European Food Safety Authority. Scientific Opinion on Dietary Reference Values for energy. *EFSA J.* **2013**, *11*, 3005, doi:10.2903/j.efsa.2013.3005.
